# Transient foot drop following motor-sparing regional anesthesia technique in total knee arthroplasty: a case report

**DOI:** 10.1093/jscr/rjag537

**Published:** 2026-07-07

**Authors:** Yan Jing Sherrie Swee, Anusha Kannan, Chi Ho Chan

**Affiliations:** Sengkang General Hospital, 110 Sengkang E Wy, Singapore 544886; Sengkang General Hospital, 110 Sengkang E Wy, Singapore 544886; Sengkang General Hospital, 110 Sengkang E Wy, Singapore 544886

**Keywords:** foot drop, regional anesthesia, motor-sparing, knee arthroplasty

## Abstract

Regional anesthesia provides excellent analgesia for total knee arthroplasty (TKA). Foot drop following motor-sparing regional anesthesia techniques is uncommon and typically transient, but it may obscure symptoms and potentially delay the diagnosis and treatment of peroneal nerve injury following TKA. We present a case of transient foot drop following an Infiltration between Popliteal Artery and Capsule of the Knee (iPACK) block in a 69-year-old female who underwent an elective TKA for her tricompartmental osteoarthritis. Although the iPACK block is generally considered motor-sparing, this case highlights a potential temporary neurological complication.

## Introduction

Pain following total knee arthroplasty (TKA) is clinically significant, hindering early mobilization and contributing to the development of chronic pain [1, 2]. Regional anesthesia provides effective perioperative analgesia, and motor-sparing techniques facilitate early and safe mobilization [3]. However, symptoms of common peroneal nerve (CPN) palsy, a serious complication of TKA with a reported incidence of 0.3%–1.3%, may be masked by regional anesthesia, potentially delaying diagnosis and treatment [4]. We present the case of a 69-year-old female who developed transient foot drop following TKA with distal femoral triangle (d-FT) and the infiltration between popliteal artery and capsule of the knee (iPACK) blocks and discuss the potential underlying causes along with strategies for prevention.

## Case description

A 69-year-old Chinese female (height 153 cm, weight 57.5 kg, body mass index 24.6 kg/m^2^) was admitted for elective left TKA for tricompartmental osteoarthritis. Her medical history includes hypertension and hyperlipidemia. She had previously undergone an uneventful right TKA under spinal anesthesia 4 years ago.

She underwent left TKA under spinal anesthesia. Spinal anesthesia was performed in the right lateral decubitus position using 2.3 ml of 0.5% hyperbaric bupivacaine and 15 μg fentanyl. Subsequently, a d-FT block and p-iPACK block were performed uneventfully in the supine position using 15 ml of 0.25% ropivacaine and 20 ml of 0.2% ropivacaine, respectively.

The surgery was carried out uneventfully and was completed in 1 h and 45 min. A thigh tourniquet was applied at 280 mmHg for the duration of the surgery. Intra-operatively, peri-articular injections were administered comprising of a total of 20 ml of 0.5% bupivacaine with adrenaline (1:200000), 10 mg of morphine, 50 mg of triamcinolone, and 30 mg of ketorolac, diluted with normal saline to a total volume of 57 ml.

Postoperatively, the patient developed a new-onset left-sided foot drop despite resolution of spinal anesthesia, which resolved spontaneously by the following morning. Throughout, she remained vascularly intact, with palpable dorsalis pedis and posterior tibial pulses. Pain was well-controlled with simple analgesia, requiring only a single 25 mg dose of oral tramadol on post-operative day (POD) one for breakthrough pain. Following the resolution of her foot drop, she fully participated in physiotherapy, progressed well with rehabilitation, and was discharged home on POD three.

## Discussion

Foot drop has been reported following motor-sparing regional anesthesia techniques such as the adductor canal block (ACB) and the iPACK block. In a retrospective review, Biehl *et al.* reported postoperative foot drop in 2 of 267 patients receiving ACB alone, and 4 of 166 patients receiving combined ACB and iPACK block, all of which resolved by POD two [5]. However, the specific ACB and iPACK technique used was not documented. Ruggiero *et al.* and Sreckovic *et al.* each reported a case of transient postoperative foot drop following a p-iPACK block, the latter in combination with a proximal ACB (p-ACB) [6, 7]. In our case, our patient experienced transient foot drop after combining d-FT and p-iPACK blocks.

**Figure 1:**
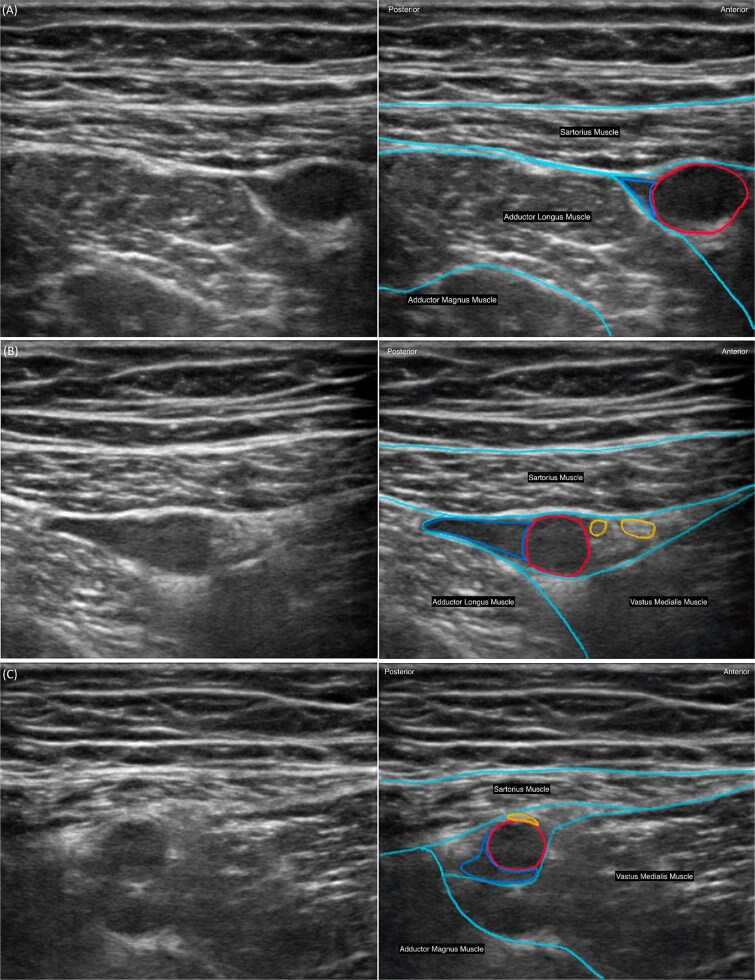
Ultrasound image of the adductor canal blocks. (A) Distal femoral triangle, the apex of the femoral triangle recognized by the point at which the medial border of the sartorius muscle crosses the medial border of the adductor longus muscle. (B) Proximal adductor canal, immediately distal to the distal femoral triangle, within the subsartorial aponeurotic compartment of the adductor canal. (C) Distal adductor canal, just proximal to the adductor hiatus, where the femoral artery exits the canal and becomes the popliteal artery. The femoral artery (red) and femoral vein (blue) are highlighted, with muscle borders outlined in cyan. Relevant muscles and bony structures are labeled.

ACB is increasingly used as a motor-sparing alternative to the femoral nerve block, providing analgesia to the anterior knee capsule. However, the anatomy of the adductor canal has only recently been better defined, distinguishing the location of the d-FT, p-ACB, and distal ACB (d-ACB) block (Fig. 1) [8, 9]. While d-FT block and p-ACB do not appear to result in local anesthetic (LA) spread through the adductor hiatus into the popliteal fossa [10, 11], d-ACB may permit such spread to the popliteal plexus [10, 12, 13]. A cadaveric dye study by Runge *et al.* demonstrated that d-ACB resulted in dye spread through the adductor hiatus into the popliteal plexus, with sciatic nerve staining observed in 10% of cases after injecting 10 ml of dye into the distal adductor canal [12]. Similarly, Gautier *et al.* performed d-ACB using 20 ml of 1% mepivacaine and reported diminished or absent sensation in the CPN and tibial nerve territories in 40% and 60% of patients, respectively [13]. These findings suggest that d-ACB may involve the CPN, potentially resulting in postoperative foot drop.

**Figure 2:**
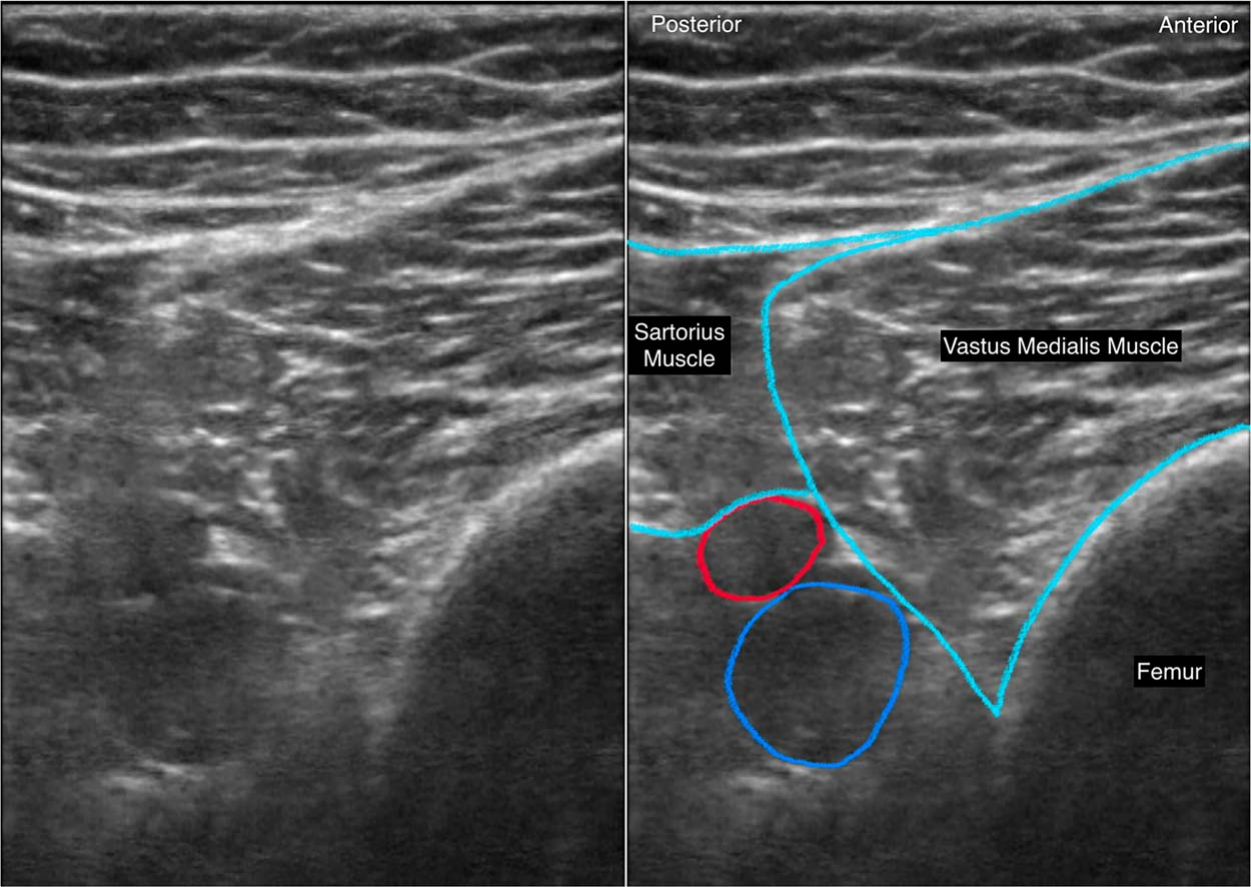
Ultrasound image of the anatomical region targeted in the proximal approach to infiltration between the popliteal artery and the capsule of the knee (p-iPACK) block. The ultrasound probe is placed transversely over the anteromedial thigh, ~1 fingerbreadth proximal to the base of the patella, and the block needle is inserted in the medial-to-lateral direction of which LA is injected into the interspace between the femur and the popliteal fossa. The femoral artery (red) and femoral vein (blue) are highlighted, with muscle borders outlined in cyan. Relevant muscles and bony structures are labeled.

**Figure 3:**
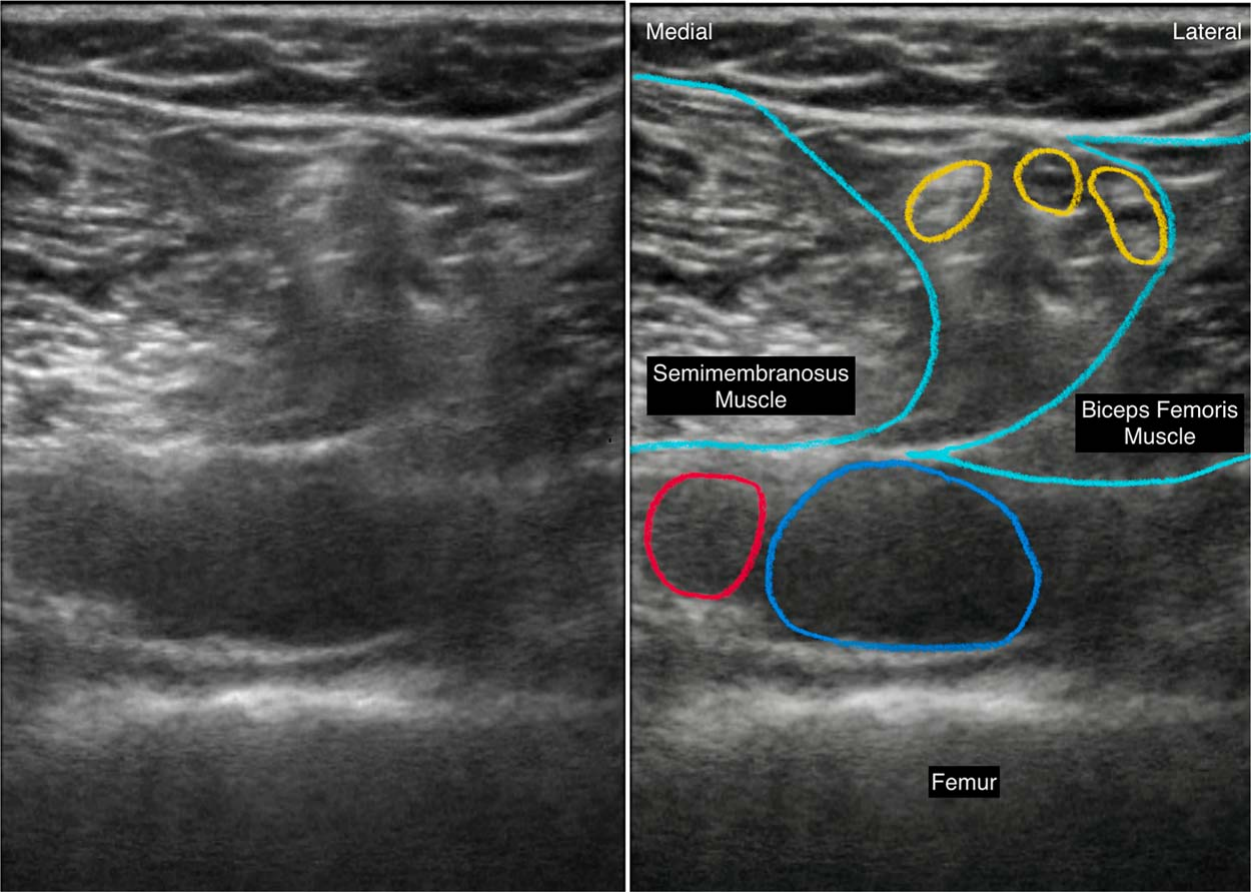
Ultrasound image of the anatomical region targeted in the distal approach to infiltration between the popliteal artery and the capsule of the knee (d-iPACK) block. The ultrasound probe is placed posteriorly over the popliteal fossa, just proximal to the femoral condyles. The popliteal artery (red) and popliteal vein (blue) are highlighted, with muscle borders outlined in cyan. Relevant muscles and bony structures are labeled.

The iPACK block has gained popularity as a motor-sparing regional anesthesia technique for TKA to provide anesthesia to the posterior knee capsule [14]. The iPACK block may be performed using two distinct approaches: the p-iPACK and distal injection technique of iPACK (d-iPACK), shown in Figs 2 and 3, respectively. In the reported cases of postoperative foot drop, when specified, the iPACK block associated with such complication appeared to involve the p-iPACK block [6, 7]. In a cadaveric study on 10 specimens, Niesen *et al.* performed a d-iPACK block using 20 ml of dye and reported no staining of the sciatic nerve or CPN [15]. A possible explanation is the difference in needle trajectory. In the p-iPACK technique, the needle is typically advanced in an anteroposterior direction, potentially facilitating posterior spread of LA towards the sciatic nerve and its branches. In contrast, the d-iPACK technique involves injection parallel to the distal femur neck, which may limit posterior spread. Nonetheless, the d-iPACK block may be the preferred iPACK block technique for minimizing the risk of inadvertent CPN block.

Although inadvertent blockade of the CPN is rare, its potential impact on patient safety warrants careful consideration. In our case, although the d-FT block may contribute to quadriceps weakness, it is unlikely to account for the postoperative foot drop observed. The cause of the transient foot drop was most likely due to CPN from the p-iPACK block. We recommend using a d-FT block or p-ACB for anterior knee capsule analgesia and a d-iPACK block for posterior capsule analgesia to minimize CPN block. In addition, limiting the volume of LA may reduce the risk of inadvertent spread, while using a lower concentration (e.g. 0.2–0.3% ropivacaine or equivalent) may help preserve motor function should such spread occur.

In conclusion, while motor-sparing regional anesthesia techniques are commonly employed for TKA, they may carry a rare risk of motor blockade of the CPN, potentially predisposing patients to postoperative foot drop. Careful selection and execution of block techniques may help mitigate the risk and reduce the incidence of such complications.
